# Elevation of high sensitive troponin T after CMR stress testing

**DOI:** 10.1186/1532-429X-13-S1-P96

**Published:** 2011-02-02

**Authors:** Mani Farazandeh, Holger C Eberle, Christoph J Jensen, Daniel John, Thomas Schlosser, Kai Nassenstein, Christoph K Naber, Georg V Sabin, Oliver Bruder

**Affiliations:** 1 Elisabeth Hospital, Essen, Germany; 2 University Hospital, Essen, Germany

## Introduction

High sensitive troponin T (hsTnT) is a highly specific marker of myocardial injury and one of the keystones in the diagnosis of acute coronary syndrome. Although hsTnT is thought to be released by myocardial cell damage, recent studies reported elevated levels after exercise testing and endurance sports. The exact mechanism of this phenomenon is discussed controversially. CMR stress testing is widely used for detection of myocardial ischemia. The kinetic of troponin release after CMR stress testing has not been studied previously.

## Purpose

To assess the impact of adenosine and dobutamine CMR stress testing on hsTnT.

## Methods

21 Patients underwent CMR stress testing (1.5 T Magnetom Avanto, Siemens Healthcare Erlangen, Germany) for evaluation of coronary artery disease using dobutamine wall motion (13 patients) or adenosine perfusion imaging (8 patients). Standard protocols included dobutamine infusion (up to 40 ug/kg/min, up to 1 mg atropine) to reach a target heart rate [(220-age)*0.85] or adenosine infusion (0.14 mg/kg/min). Blood samples were taken immediately before and 12-24 hours after CMR.

## Results

At baseline, hsTnT levels of all patients were below the detection limit of 0.010 ng/ml. After dobutamine stress, hsTnT levels raised in 7 patients (54%), of which 3 had evidence of myocardial ischemia in wall motion analysis. 4 patients with an increase in hsTnT did not show ischemia in CMR. Elevation of hsTnT was not detected in any patient after adenosine stress. A significant positive correlation between hsTnT levels and increase in systolic blood pressure after dobutamine stress was observed (0.554, p<0.05). Myocardial ischemia (1 in the adenosine group, 5 in the dobutamine group) was not associated with the release of hsTnT.

## Conclusions

HsTnT release seems to be a common phenomenon using different stress testing modalities associated with increased blood pressures. This is supported by previous reports of hsTnT elevation related to increased blood pressure like physical stress testing, whereas adenosine does not alter blood pressure. Therefore, mechanisms different from cardiomyocyte necrosis might contribute to hsTnT release.

